# Third trimester anemia extends the length of hospital stay after delivery

**DOI:** 10.4274/tjod.87864

**Published:** 2017-09-30

**Authors:** Kazibe Koyuncu, Batuhan Turgay, Yavuz Emre Şükür, Bircan Yıldırım, Can Ateş, Feride Söylemez

**Affiliations:** 1 Ankara University Faculty of Medicine, Department of Obstetrics and Gynecology, Ankara, Turkey

**Keywords:** Anemia, hospitalization, third trimester

## Abstract

**Objective:**

To assess the relationship between maternal third trimester anemia and hospital stay after delivery.

**Materials and Methods:**

In this retrospective cross-sectional study, 695 women aged 18-42 years were included between January 2016 and June 2016. Obstetric outcomes and fetal outcomes were measured. Statistical analysis was performed using SPSS, version 19.0 (SPSS, Chicago, Illinois).

**Results:**

The prevalence of anemia in this study was 15.2%. The study population was divided into three groups according to hemoglobin (Hb) levels. Group 1 consisted of patients with Hb <8.5 g/dL, group 2 Hb 8.5-11 g/dL, and group 3 Hb >11 g/dL. Higher levels of Hb were associated with shorter stay in hospital (p=0.028). In binary comparison, no significant difference was observed between groups 2 and 3, whereas it was statistically different from group 1. Fetal weight (p=0.562), neonatal intensive care unit admission (p=0.596), APGAR score 1^st^ (p=0.674) and 5^th^ minute (p=0.876), type of delivery (p=0.831), and gestational age (p=0.798) were not statistically different between the groups; however, hospitalization time was significantly different (p=0.028).

**Conclusion:**

Maternal anemia in the third trimester prolongs hospitalization time after delivery. Anemia effects pregnancy and the fetus in the postpartum period in addition to the prenatal period.

## PRECIS:

In this study, we have assessed the relationship between maternal third trimester anemia and hospital stay after delivery.

## INTRODUCTION

Maternal anemia remains an important public health problem in under-developed and developing countries like ours. The Centers for Disease Control and Prevention suggested that anemia should be defined as hemoglobin levels of less than 11 g/dL (hematocrit less than 33%) in the first and third trimesters and less than 10.5 g/dL (hematocrit less than 32%) in the second trimester^(1)^. The prevalence of anemia among pregnant women seems to differ between countries and even between regions of a country. It is estimated that half of all pregnant worldwide women are anemic^(2)^. The anemia prevalence in different regions of Turkey were reported between 27% and 88%, on an average 50%^(3)^.

Anemia is a physiologic result of pregnancy, and to some extent, it may be necessary. The absence of physiologic anemia has been suggested to be associated with stillbirth^(4,5)^. Maternal anemia may have a significant impact on fetal outcome in terms of preterm birth and infant deaths^(6)^. The relationship between low birth weight, preterm birth, small for gestational age, perinatal mortality, neonatal mortality, gestational diabetes, preeclampsia, and mode of delivery according to maternal anemia status has been shown in the literature, yet it is not precisely concluded(4). However, the impact of severity of anemia on obstetric outcomes is controversial. Several studies suggest that only severe anemia leads to poor obstetrics outcomes^(7).^

The aim of our study was to identify the impact of third trimester hemoglobin values on perinatal outcomes and type and time of delivery.

## MATERIALS AND METHODS

The retrospective cross-sectional study was conducted in the obstetrics out-patient clinic in a university-based hospital between January 2016 and April 2016. All data of women who were referred for routine third trimester follow-up were recorded. The inclusion criteria were as follows: age between 18 and 45 years, singleton pregnancy, iron supplementation started around 18^th^ gestational week, gestational age between 28 and 40 weeks during the study period, and at least one complete blood count during the third trimester. The exclusion criteria were history of preterm delivery, elevated risk pregnancy, previous uterine surgery including cesarean section, multiple gestation, any type of chronic systemic disease, and not receiving iron supplementation.

All analyzed data were obtained from the patients’ charts and medical records. Patients were divided into three groups: group 1 consisted of patients with Hb <8.5 g/dL, group 2 comprised patients with Hb 8.5- 11 g/dL, and group 3 constituted patients with Hb >11 g/dL. The main outcome measures were perinatal and neonatal outcome parameters such as gestational age at delivery, mode of delivery, birth weight and APGAR scores, type of delivery, and duration of hospitalization. Ethics committee approval and informed consent were not taken as it is a retrospective study.

### Statistical Analysis

Statistical analysis was performed using SPSS, version 19.0 (SPSS, Chicago, Illinois). Due to the normal distribution of all data, ANOVA test was used for the determination of differences between the three groups. The independent sample t-test was used for to determine differences between the two sexes. Categorical data were assessed using the chi-square test. P<0.05 was considered statistically significant.

## RESULTS

A total of 695 pregnant women were assessed for eligibility. Among those, 265 patients were excluded due to being lost to follow-up (n=97), multiple pregnancy (n=13), not receiving iron supplementation (n=92), presence of chronic disease (n=73). As a result, 433 patients were included in the final analyses. The mean third trimester hemoglobin level was 11.98±5.44 mg/dL (7-15.2 mg/dL). Anemia prevalence in our study population was 15.2%. The characteristics of the study population are presented in Table 1.

Table 2 represents the comparison of perinatal outcome measures between groups 1, 2, and 3. The only parameter that showed a statistically significant difference between the groups was the duration of hospitalization. The duration of hospitalization was significantly longer in group 1 than in groups 2 and 3 (p=0.028).

## DISCUSSION

The aim of our study was to identify the impact of third trimester hemoglobin values on perinatal outcomes and type and time of delivery. According to the results, severe anemia during the third trimester results in increased duration of hospitalization. Otherwise, it has no adverse effects on outcomes.

Anemia during the third trimester of pregnancy is defined as Hb less than 11 g/dL or hematocrit less than 33% by the CDC^(1)^ and its prevalence can differ between populations and geographic regions. In our study, the prevalence of anemia was found as 15.2% during the third trimester of pregnancy. Although different anemia prevalences have been reported from various parts of our country, the prevalence of third trimester anemia in our study was similar to that reported from a large maternity hospital in our city^(7)^. Both the prevalence from our study and the other study seem to be lower than other parts of the country. This situation might be due to higher socioeconomic status resulting in appropriate follow-up during pregnancy and proper iron replacement therapy. Furthermore, grand-multiparity was rare in our study population and multiparity is also a risk factor for anemia in pregnant women^(8)^.

According to the results obtained from our study, severe third trimester anemia is associated with longer hospital stay after delivery. The main reason for longer hospitalization is postpartum anemia treatment, either by transfusion or intravenous iron supplementation^(9)^. Lin et al.^(10)^ reported that every 1-U decrease in hemoglobin level extended the median length of hospital stay by 1.5 days among anemic general medical inpatients. Also, it has been reported that anemia increased the 30-day unplanned readmission rate in addition to extending hospital stay^(11)^.

Iron supplementation may be preventive for adverse effects of anemia^(12,13)^. A recent Cochrane review Peña-Rosas et al.^(13)^ concluded that women with anemia were at greater risk of having low-birth-weight infants. On the contrary, Scholl et al.^(14)^ suggested a subdivision of anemia according to etiology and gestational age and reported that third trimester anemia due to iron deficiency had no correlation with low birth weight. In addition, some other studies concluded that after adjusting for confounders, anemia was not relevant to poor neonatal outcomes because these relationships were a result of poverty or low socioeconomic status^(14)^. In our study, we also failed to show a significant impact of anemia on neonatal outcomes such as birth weight, APGAR scores at first and fifth minutes, and neonatal intensive care unit admission. As local clinical policy, we clamp the cord 1-3 minutes after birth and this may be the reason for the neonatal results obtained from our study.

Sehgal et al.^(15)^ reported that anemia did not affect the mode of delivery in nulliparous pregnant women. Mild anemia seems to be inconclusive for estimating mode of delivery^(15)^. Van Bogaert^(16)^ also suggested no effect of anemia during first trimester of pregnancy on the mode of delivery. Our results were also in accordance with these studies. In the present study, we excluded patients with previous uterine surgery to identify the effect of anemia on the mode of delivery. However, we found no significant effect of anemia on the mode of delivery. In addition, the indications for cesarean section did not differ between the groups.

One of the strengths of our study was the standardized follow-up and management protocol for each patient. Also, neonatal results were obtained from a single center. The strict inclusion and exclusion criteria to create a homogenous study population add credence to our results.

### Study Limitations

The main limitations of our study were the retrospective design and small number of patients included. However, the present study was conducted in a tertiary referral center and we excluded high-risk patients. Hence, it was not possible to include more patients in such a study using strict inclusion criteria, conducted in a single center.

## CONCLUSION

Anemia during the third trimester extends the length of hospital stay in the postpartum period. However, it does not seem to affect neonatal outcomes and mode of delivery. Prevention and/or treatment of anemia before delivery can shorten the duration of hospital stay. Prospective studies in large cohorts are needed to clarify the exact effects of mild and severe anemia on perinatal and neonatal outcomes.

## Figures and Tables

**Table 1:**
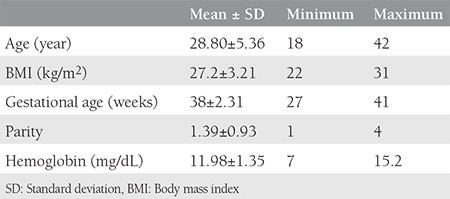
Characteristics of the study population

**Table 2:**
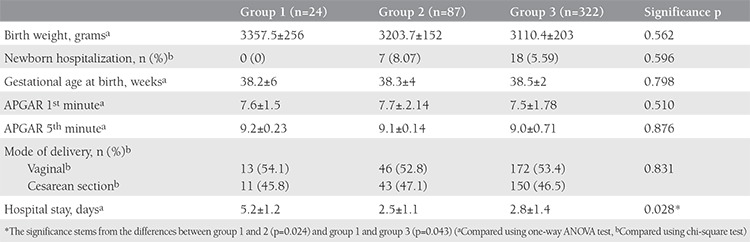
Comparison of hemoglobin values between the three groups (maternal third trimester hemoglobin level groups as lower 8.5 mg/dL, between 8.5 and 11 mg/dL, upper 11 mg/dL)
